# 2-Methyl-4,6-bis­(1-methyl­hydrazino)pyrimidine

**DOI:** 10.1107/S1600536809021643

**Published:** 2009-06-10

**Authors:** Daniel J. Hutchinson, Lyall R. Hanton, Stephen C. Moratti

**Affiliations:** a Department of Chemistry, University of Otago, PO Box 56, Dunedin, New Zealand

## Abstract

In the title compound, C_7_H_14_N_6_, the amine groups of the two methyl­hydrazino substituents are orientated in the opposite direction to the methyl substituent at the 2-position of the pyrimidine ring. The mol­ecule is almost planar with only the two amine N atoms lying substanti­ally out of the mean plane of the pyrimidine ring [by 0.1430 (2) and 0.3092 (2) Å]. The H atoms on these amine groups point inwards towards the aromatic ring, such that the lone pair of electrons points outwards from the mol­ecule. Each mol­ecule is linked to two others through N—H⋯N hydrogen bonds between the two amino groups, forming a one-dimensional chain in the [010] direction. Offset face-to-face π–π stacking inter­actions between the pyrimidine rings organize these chains into a two-dimensional array [centroid–centroid distance = 3.789 (2) Å].

## Related literature

For the use of related compounds in the synthesis of mol­ecular strands see: Schmitt *et al.* (2003 ▶), Schmitt & Lehn (2003 ▶), Gardinier *et al.* (2000 ▶).
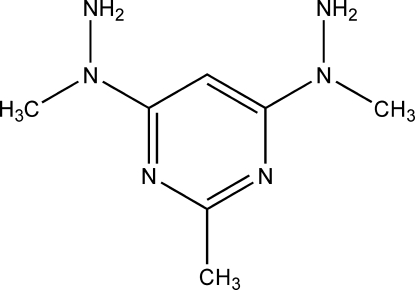


## Experimental

### 

#### Crystal data

C_7_H_14_N_6_
*M* *_r_* = 182.24
Monoclinic, 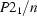

*a* = 9.2255 (6) Å
*b* = 8.5075 (6) Å
*c* = 12.2323 (7) Å
β = 109.233 (3)°
*V* = 906.48 (10) Å^3^
*Z* = 4
Mo *K*α radiation
μ = 0.09 mm^−1^
*T* = 90 K
0.40 × 0.32 × 0.18 mm

#### Data collection

Bruker APEXII CCD area-detector diffractometer
Absorption correction: multi-scan (*SADABS*; Bruker, 2006 ▶) *T*_min_ = 0.905, *T*_max_ = 0.980
16072 measured reflections
1691 independent reflections
1652 reflections with *I* > 2σ(*I*)
*R*_int_ = 0.026

#### Refinement

*R*[*F* ^2^ > 2σ(*F* ^2^)] = 0.035
*wR*(*F* ^2^) = 0.096
*S* = 1.04
1691 reflections
133 parameters
H atoms treated by a mixture of independent and constrained refinement
Δρ_max_ = 0.20 e Å^−3^
Δρ_min_ = −0.21 e Å^−3^

### 

Data collection: *APEX2* (Bruker, 2006 ▶); cell refinement: *APEX2* and *SAINT* (Bruker 2006 ▶); data reduction: *SAINT*; program(s) used to solve structure: *SIR97* (Altomare *et al.*, 1999 ▶); program(s) used to refine structure: *SHELXL97* (Sheldrick, 2008 ▶); molecular graphics: *SHELXTL* (Sheldrick, 2008 ▶) and *Mercury* (Bruno *et al.*, 2002 ▶); software used to prepare material for publication: *SHELXTL* and *enCIFer* (Allen *et al.*, 2004 ▶).

## Supplementary Material

Crystal structure: contains datablocks global, I. DOI: 10.1107/S1600536809021643/bv2120sup1.cif

Structure factors: contains datablocks I. DOI: 10.1107/S1600536809021643/bv2120Isup2.hkl

Additional supplementary materials:  crystallographic information; 3D view; checkCIF report

## Figures and Tables

**Table 1:** Hydrogen-bond geometry (Å, °)

*D*—H⋯*A*	*D*—H	H⋯*A*	*D*⋯*A*	*D*—H⋯*A*
N6—H61⋯N4^i^	0.932 (16)	2.177 (17)	3.0933 (18)	167.5 (14)
N4—H4*B*⋯N2^ii^	0.909 (16)	2.504 (16)	3.3319 (18)	151.6 (12)
N6—H62⋯N1^ii^	0.945 (17)	2.418 (17)	3.334 (2)	163.3 (13)
N4—H4*A*⋯N2^iii^	0.930 (16)	2.268 (16)	3.1722 (18)	164.2 (13)

Symmetry codes: (i) 
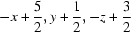
; (ii) 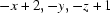; (iii) 
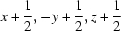
.

**Fig. 1.**
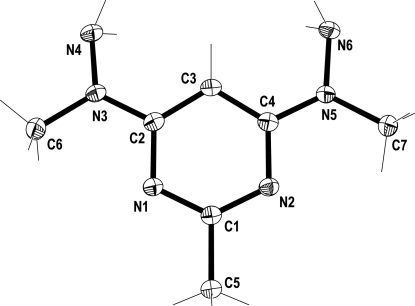
The asymmetric unit of (1) showing the atom numbering with displacement ellipsoids drawn at the 50% probability level.

**Fig. 2.**
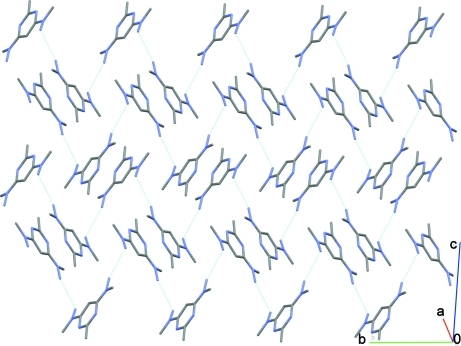
View of the arrangement of 1 into one-dimensional chains through H-bonding and organization of the chains into a two-dimensional array through π-π stacking.

## supplementary crystallographic information

### Comment

Although 1 is unstable in air, we were able to isolate X-ray quality crystals. Compound 1 was prepared in quantitative yield through the reaction of methylhydrazine and 4,6-dichloro-2-methylpyrimidine under an inert N_2_ atmosphere. In 1 the amine groups (N4 and N6) were orientated in the opposite direction to the methyl group (C5). The molecule was planar with only N4 and N6 being out of the mean plane of the pyrimidine ring by 0.1430 (2) and 0.3092 (2) Å, respectively. The hydrogen atoms on these amine groups were located from difference Fourier maps and freely refined. They pointed inwards towards C3 such that the lone pair of electrons on N4 and N6 pointed outwards from the molecule.

Each molecule of 1 was linked to two others through H-bonding between N4 and N6 (Figure 2). The N6—H···N4 distance was measured as 2.177 (16) Å, which corresponded to a N6···N4 distance of 3.093 (2) Å. This H-bond linked molecules of 1 together to form a one dimensional chain in the [0 1 -1] direction. The angle between the planes of adjacent H-bonded molecules was 72.97 (1)°. Offset, face-to-face π-π stacking interactions between the pyrimidine rings organized these chains into a two dimensional array. The centroid to centroid distance for this π-π interaction was 3.789 (2) Å.

### Experimental

Under magnetic stirring, 4,6-dichloro-2-methylpyrimidine, (0.4792 g, 2.94 mmol) dissolved in EtOH (30 ml), was added by portions over 20 min to ice cooled methylhydrazine (2.00 ml, 38.0 mmol) flushed with Ar. The mixture was refluxed for 6 h under an inert atmosphere of N_2_. After cooling, residual methylhydrazine and EtOH were evaporated, K_2_CO_3_ (1.025 g) and CHCl_3_ (50 ml) were added to the solid residue, and the mixture was stirred for 20 min. The liquid phase was filtered and the solid was washed with more CHCl_3_ (50 ml then 30 ml). The combined liquid fractions were evaporated and the resulting solid was dried *in vacuo* to gave 1 as a white solid (0.5566 g, quant.), unstable in air: ^1^H NMR (CDCl_3_, 500 MHz) δ/p.p.m.: 5.95 (1*H*, s, H5), 3.99 (4*H*, bs, NH_2_), 3.22 (6*H*, s, H8), 2.37 (3*H*, s, H7). ^13^C NMR (CDCl_3_, 500 MHz) δ/p.p.m.: 166.0 (C2), 165.2 (C4, C6), 78.1 (C5), 39.9 (C8), 26.2 (C7). ESMS m/*z* Found: 365.1063 [2*M*+H]^+^, 183.1353 [*M*+H]^+^, 151.0966 [*M*-(NH_2_)_2_]^+^. Calc. for C_7_H_14_N_6_: [*M*+H]^+^ 183.1353. Selected IR (KBr disc) ν/cm^-1^: 3296 (s, NH str), 3177 (m, CH str), 2933 (m, CH str), 1588 (s, br, NH bend), 1499 (m, pym str), 1399 (m, CH bend), 1136 (w, CN str). Crystals suitable for X-ray determination were grown by slow evaporation of a CDCl_3_ solution of 1.

### Refinement

All H-atoms bound to carbon were refined using a riding model with d(C—H) = 0.93 Å, *U*_iso_=1.2*U*_eq_ (C) for the CH H atoms and d(C—H) = 0.96 Å, *U*_iso_=1.5*U*_eq_ (C) for the CH_3_ H atoms. All H-atoms bound to nitrogen were located from difference Fourier maps and freely refined with *U*_iso_=1.5*U*_eq_ (N).

### Crystal data

**Table:**

C_7_H_14_N_6_	*F*(000) = 392
*M**_r_* = 182.24	*D*_x_ = 1.335 Mg m^−^^3^
Monoclinic, *P*2_1_/*n*	Mo *K*α radiation, λ = 0.71069 Å
Hall symbol: -P 2yn	Cell parameters from 6739 reflections
*a* = 9.2255 (6) Å	θ = 2.4–39.2°
*b* = 8.5075 (6) Å	µ = 0.09 mm^−^^1^
*c* = 12.2323 (7) Å	*T* = 90 K
β = 109.233 (3)°	Rhomb, colourless
*V* = 906.48 (10) Å^3^	0.40 × 0.32 × 0.18 mm
*Z* = 4	

### Data collection

**Table:**

Bruker APEXII CCD area-detector diffractometer	1691 independent reflections
Radiation source: fine-focus sealed tube	1652 reflections with *I* > 2σ(*I*)
graphite	*R*_int_ = 0.026
φ and ω scans	θ_max_ = 25.5°, θ_min_ = 3.0°
Absorption correction: multi-scan (*SADABS*; Bruker, 2006)	*h* = −11→11
*T*_min_ = 0.905, *T*_max_ = 0.980	*k* = −10→10
16072 measured reflections	*l* = −14→14

### Refinement

**Table:**

Refinement on *F*^2^	Primary atom site location: structure-invariant direct methods
Least-squares matrix: full	Secondary atom site location: difference Fourier map
*R*[*F*^2^ > 2σ(*F*^2^)] = 0.035	Hydrogen site location: inferred from neighbouring sites
*wR*(*F*^2^) = 0.096	H atoms treated by a mixture of independent and constrained refinement
*S* = 1.04	*w* = 1/[σ^2^(*F*_o_^2^) + (0.0521*P*)^2^ + 0.3928*P*] where *P* = (*F*_o_^2^ + 2*F*_c_^2^)/3
1691 reflections	(Δ/σ)_max_ < 0.001
133 parameters	Δρ_max_ = 0.20 e Å^−^^3^
0 restraints	Δρ_min_ = −0.21 e Å^−^^3^

### Special details

**Table:**

Geometry. All e.s.d.'s (except the e.s.d. in the dihedral angle between two l.s. planes) are estimated using the full covariance matrix. The cell e.s.d.'s are taken into account individually in the estimation of e.s.d.'s in distances, angles and torsion angles; correlations between e.s.d.'s in cell parameters are only used when they are defined by crystal symmetry. An approximate (isotropic) treatment of cell e.s.d.'s is used for estimating e.s.d.'s involving l.s. planes.
Refinement. Refinement of *F*^2^ against ALL reflections. The weighted *R*-factor *wR* and goodness of fit *S* are based on *F*^2^, conventional *R*-factors *R* are based on *F*, with *F* set to zero for negative *F*^2^. The threshold expression of *F*^2^ > σ(*F*^2^) is used only for calculating *R*-factors(gt) *etc*. and is not relevant to the choice of reflections for refinement. *R*-factors based on *F*^2^ are statistically about twice as large as those based on *F*, and *R*- factors based on ALL data will be even larger.

### Fractional atomic coordinates and isotropic or equivalent isotropic displacement parameters (Å^2^)

**Table:**

	*x*	*y*	*z*	*U*_iso_*/*U*_eq_	
C1	0.73886 (13)	0.17687 (13)	0.47904 (9)	0.0164 (3)	
C2	0.93621 (12)	0.07975 (13)	0.63049 (9)	0.0154 (3)	
C3	1.04186 (12)	0.15748 (13)	0.59004 (9)	0.0161 (3)	
H3	1.1469	0.1510	0.6289	0.019*	
C4	0.98251 (13)	0.24539 (13)	0.48866 (10)	0.0157 (3)	
C5	0.56857 (13)	0.18783 (15)	0.41657 (10)	0.0222 (3)	
H5A	0.5198	0.0927	0.4284	0.033*	
H5B	0.5501	0.2024	0.3353	0.033*	
H5C	0.5273	0.2754	0.4462	0.033*	
C6	0.87526 (13)	−0.07473 (15)	0.78187 (10)	0.0208 (3)	
H6A	0.8765	−0.0070	0.8449	0.031*	
H6B	0.9064	−0.1786	0.8108	0.031*	
H6C	0.7734	−0.0785	0.7268	0.031*	
C7	1.01177 (14)	0.42150 (15)	0.33820 (11)	0.0233 (3)	
H7A	0.9543	0.3559	0.2749	0.035*	
H7B	1.0940	0.4712	0.3194	0.035*	
H7C	0.9454	0.5004	0.3519	0.035*	
N1	0.78212 (10)	0.08954 (11)	0.57463 (8)	0.0164 (2)	
N2	0.82871 (11)	0.25598 (11)	0.43155 (8)	0.0167 (2)	
N3	0.98058 (10)	−0.01433 (12)	0.72595 (8)	0.0186 (2)	
N4	1.13791 (11)	−0.04093 (12)	0.78658 (8)	0.0186 (2)	
H4A	1.1891 (17)	0.0522 (19)	0.8151 (13)	0.028*	
H4B	1.1821 (17)	−0.0851 (19)	0.7376 (14)	0.028*	
N5	1.07451 (11)	0.32649 (12)	0.44126 (8)	0.0196 (2)	
N6	1.23407 (11)	0.29663 (13)	0.47455 (9)	0.0202 (2)	
H61	1.2826 (18)	0.3324 (19)	0.5497 (14)	0.030*	
H62	1.2505 (17)	0.187 (2)	0.4720 (13)	0.030*	

### Atomic displacement parameters (Å^2^)

**Table:**

	*U*^11^	*U*^22^	*U*^33^	*U*^12^	*U*^13^	*U*^23^
C1	0.0154 (6)	0.0164 (5)	0.0172 (6)	0.0002 (4)	0.0053 (4)	−0.0020 (4)
C2	0.0170 (5)	0.0143 (5)	0.0147 (5)	0.0007 (4)	0.0048 (4)	−0.0043 (4)
C3	0.0129 (5)	0.0173 (5)	0.0171 (5)	−0.0002 (4)	0.0035 (4)	−0.0027 (4)
C4	0.0156 (5)	0.0142 (5)	0.0175 (5)	−0.0010 (4)	0.0059 (4)	−0.0041 (4)
C5	0.0146 (6)	0.0280 (7)	0.0226 (6)	−0.0004 (5)	0.0044 (5)	0.0059 (5)
C6	0.0194 (6)	0.0255 (6)	0.0187 (6)	0.0002 (5)	0.0078 (5)	0.0020 (5)
C7	0.0199 (6)	0.0246 (6)	0.0265 (6)	0.0002 (5)	0.0090 (5)	0.0065 (5)
N1	0.0142 (5)	0.0180 (5)	0.0171 (5)	0.0004 (4)	0.0052 (4)	−0.0008 (4)
N2	0.0144 (5)	0.0179 (5)	0.0174 (5)	−0.0002 (4)	0.0047 (4)	−0.0002 (4)
N3	0.0135 (5)	0.0244 (5)	0.0172 (5)	0.0009 (4)	0.0040 (4)	0.0036 (4)
N4	0.0146 (5)	0.0213 (5)	0.0179 (5)	0.0020 (4)	0.0026 (4)	0.0000 (4)
N5	0.0121 (5)	0.0238 (5)	0.0228 (5)	−0.0001 (4)	0.0056 (4)	0.0035 (4)
N6	0.0138 (5)	0.0238 (6)	0.0226 (5)	−0.0011 (4)	0.0056 (4)	−0.0020 (4)

### Geometric parameters (Å, °)

**Table:**

C1—N1	1.3308 (15)	C6—H6A	0.9600
C1—N2	1.3397 (15)	C6—H6B	0.9600
C1—C5	1.5067 (17)	C6—H6C	0.9600
C2—N1	1.3619 (16)	C7—N5	1.4480 (16)
C2—N3	1.3625 (15)	C7—H7A	0.9600
C2—C3	1.3965 (16)	C7—H7B	0.9600
C3—C4	1.3963 (17)	C7—H7C	0.9600
C3—H3	0.9300	N3—N4	1.4138 (14)
C4—N2	1.3626 (16)	N4—H4A	0.930 (16)
C4—N5	1.3626 (16)	N4—H4B	0.909 (16)
C5—H5A	0.9600	N5—N6	1.4151 (15)
C5—H5B	0.9600	N6—H61	0.932 (16)
C5—H5C	0.9600	N6—H62	0.945 (17)
C6—N3	1.4540 (15)		
			
N1—C1—N2	127.74 (10)	H6A—C6—H6C	109.5
N1—C1—C5	116.17 (10)	H6B—C6—H6C	109.5
N2—C1—C5	116.09 (10)	N5—C7—H7A	109.5
N1—C2—N3	115.80 (10)	N5—C7—H7B	109.5
N1—C2—C3	121.89 (10)	H7A—C7—H7B	109.5
N3—C2—C3	122.28 (10)	N5—C7—H7C	109.5
C4—C3—C2	116.94 (10)	H7A—C7—H7C	109.5
C4—C3—H3	121.5	H7B—C7—H7C	109.5
C2—C3—H3	121.5	C1—N1—C2	115.87 (9)
N2—C4—N5	115.97 (10)	C1—N2—C4	115.66 (10)
N2—C4—C3	121.89 (10)	C2—N3—N4	120.69 (9)
N5—C4—C3	122.15 (10)	C2—N3—C6	123.50 (10)
C1—C5—H5A	109.5	N4—N3—C6	115.24 (9)
C1—C5—H5B	109.5	N3—N4—H4A	111.5 (9)
H5A—C5—H5B	109.5	N3—N4—H4B	109.0 (9)
C1—C5—H5C	109.5	H4A—N4—H4B	108.5 (13)
H5A—C5—H5C	109.5	C4—N5—N6	121.43 (10)
H5B—C5—H5C	109.5	C4—N5—C7	121.78 (10)
N3—C6—H6A	109.5	N6—N5—C7	115.49 (9)
N3—C6—H6B	109.5	N5—N6—H61	110.0 (10)
H6A—C6—H6B	109.5	N5—N6—H62	109.3 (9)
N3—C6—H6C	109.5	H61—N6—H62	108.9 (13)

### Hydrogen-bond geometry (Å, °)

**Table:**

*D*—H···*A*	*D*—H	H···*A*	*D*···*A*	*D*—H···*A*
N6—H61···N4^i^	0.932 (16)	2.177 (17)	3.0933 (18)	167.5 (14)
N4—H4B···N2^ii^	0.909 (16)	2.504 (16)	3.3319 (18)	151.6 (12)
N6—H62···N1^ii^	0.945 (17)	2.418 (17)	3.334 (2)	163.3 (13)
N4—H4A···N2^iii^	0.930 (16)	2.268 (16)	3.1722 (18)	164.2 (13)

Symmetry codes: (i) −*x*+5/2, *y*+1/2, −*z*+3/2; (ii) −*x*+2, −*y*, −*z*+1; (iii) *x*+1/2, −*y*+1/2, *z*+1/2.
